# Influences of Annealing Treatment on Soft Magnetic Properties, Mechanical Properties and Microstructure of Fe_24.94_Co_24.94_Ni_24.94_Al_24.94_Si_0.24_ High-Entropy Alloy

**DOI:** 10.3390/e28010110

**Published:** 2026-01-16

**Authors:** Shiqi Zhang, Pin Jiang, Xuanbo Shi, Xiaohua Tan, Hui Xu

**Affiliations:** 1 State Key Laboratory of Materials for Advanced Nuclear Energy, Shanghai University, Shanghai 200444, China; zhangshiqi0320@shu.edu.cn (S.Z.); 3221659799@shu.edu.cn (P.J.); shixuanbo830@shu.edu.cn (X.S.); 2 School of Materials Science and Engineering, Shanghai University, Shanghai 200444, China

**Keywords:** high-entropy alloy, coherent precipitation, soft magnetic property, mechanical property, annealing

## Abstract

In order to meet the ever-growing demand in modern power electronics, the advanced soft magnetic materials (SMMs) are required to exhibit both excellent soft magnetic performance and mechanical properties. In this work, the effects of an annealing treatment on the soft magnetic properties, mechanical properties and microstructure of the Fe_24.94_Co_24.94_Ni_24.94_Al_24.94_Si_0.24_ high-entropy alloy (HEA) are investigated. The as-cast HEA consists of a body-centered cubic (BCC) matrix phase and spherical B2 nanoprecipitates with a diameter of approximately 5 nm, where a coherent relationship is established between the B2 phase and the BCC matrix. After annealing at 873 K, the alloy retains both the BCC and B2 phases, with their coherent relationship preserved; besides the spherical B2 nanoprecipitates, rod-shaped B2 nanoprecipitates are also observed. After the annealing treatment, the saturation magnetization (M_s_) of the alloy varies slightly within the range of 103–113 Am^2^/kg, which may be induced by the precipitation of this rod-shaped nanoprecipitate phase in the alloy. The increase in the coercivity (H_c_) of annealed HEA is due to the inhomogeneous grain distribution, increased lattice misfit and high dislocation density induced by the annealing. The nanoindentation result reveals that the hardness after annealing at 873 K exhibits a 25% improvement compared with the hardness of as-cast HEA, which is mainly due to dislocation strengthening and precipitation strengthening. This research finding can provide guidance for the development of novel ferromagnetic HEAs, so as to meet the demands for materials with excellent soft magnetic properties and superior mechanical properties in the field of sustainable electrical energy.

## 1. Introduction

High-entropy alloys (HEAs) are composed of more than five principal elements in equiatomic or near-equiatomic ratios and tend to form solid solutions with a simple crystal structure (e.g., face-centered cubic (FCC), body-centered cubic (BCC)) [1,2,3]. Moreover, the HEAs show four core effects (i.e., high-entropy effect, severe lattice distortion effect, sluggish diffusion effect and cocktail effect), leading to the exhibition of excellent mechanical properties, good corrosion resistance and wear resistance [4,5,6,7]. The ferromagnetic HEAs in particular have attracted considerable attention because they can meet the requirement of advanced soft magnetic materials (SMMs) with a combination of good soft magnetic performance and mechanical properties [8,9]. For example, the Fe–Co–Ni–Al–Si HEAs (the saturation magnetization M_s_ = 70–140 Am^2^/kg, the yield strength σ_0.2_ = 500–1500 MPa), the Fe–Co–Ni–Al–Cr HEAs (M_s_ = 80–100 Am^2^/kg, σ_0.2_ = 500–1400 MPa), the Fe–Co–Ni–Al–Cu HEAs (M_s_ = 80–135 Am^2^/kg, σ_0.2_ = 500–1500 MPa) and the Fe–Co–Ni–Al–Ti HEAs (M_s_ = 110–140 Am^2^/kg, σ_0.2_ = 600–1100 MPa) exhibit good soft magnetic and mechanical properties [10,11,12,13,14,15,16,17], which are regarded as promising candidates for novel SMMs and have potential applications in high-speed rotating motors and high loaded conditions in power electronics [18,19].

In order to further improve the magnetic and/or mechanical properties, three primary strategies are usually employed. One approach is alloying. For instance, the soft magnetic properties of the Fe–Co–Ni–Al–Cu HEAs can be enhanced by the addition of Sn, Ga, Nd and Y [20,21,22]. The mechanical properties of Fe–Co–Ni–Cr HEAs are significantly improved through the addition of Ni and Si [23,24]. The second method is the optimization of compositions of HEAs. It has been reported that the soft magnetic and mechanical properties are improved by adjusting the content of (MnAl) in the Fe–Co–Ni–Al–Mn HEAs and tuning the concentration of (AlSi) in the Fe–Co–Ni–Al–Si HEA [25,26]. The third strategy is using an annealing treatment. Khan et al. found that the mechanical properties of Fe_64_Ni_14_Cr_12_Co_4_(TiAl)_6_ HEA could be effectively improved by changing the annealing temperatures. The high ultimate tensile strength of 1640 MPa and an elongation of 15% were achieved after annealing at 873 K, which resulted from an ultrafine grain structure and transformation-induced plasticity [27]. The different annealing temperatures also affect the soft magnetic property of the Co_35_Fe_10_Mn_5_Ni_30_Ti_20_ HEA. The M_s_ of 95 Am^2^/kg and low coercivity were obtained in the annealed HEA at 973 K, which ascribed to the formation of an allotropic fcc phase with large magnetic moment value [28]. Liu et al. found that the microstructure of AlCu_0.25_CoCrFeNi_2.1_ HEA could be optimized by turning the annealing time. The precipitation of the Cu-rich B2 phase was triggered and the proportion of FCC/(BCC + B2) was changed after annealing at various times, leading to good mechanical properties with high tensile strength (991 MPa) and elongation (30%) after 8 h annealing [29]. However, as the candidates for novel SMMs, the improvement of the individual magnetic or mechanical property of ferromagnetic HEAs after annealing is not enough. Simultaneously improving magnetic and mechanical properties remains a great challenge.

Raabe et al. overcame this problem by introducing the coherent L1_2_ nanoprecipitates into the FCC matrix in the Fe–Co–Ni–Ta–Al HEA. Then, annealing treatments were employed to further increase the properties. The simultaneous improvement of soft magnetic performance and mechanical properties is achieved through tuning the size and volume of coherent nanoparticles after annealing at 1173 K for multiple hours [30,31].

In our previous work [32], compared to the equiatomic FeCoNiAl alloy, the soft magnetic and mechanical properties of as-cast Si-doped Fe_24.94_Co_24.94_Ni_24.94_Al_24.94_Si_0.24_ HEA were increased by highly coherent B2 nanoprecipitates in the BCC matrix. In this study, we investigate the influences of the annealing treatment at different temperatures on the microstructures, magnetic and mechanical properties of Fe_24.94_Co_24.94_Ni_24.94_Al_24.94_Si_0.24_ HEA. The mechanical properties are improved after annealing at 873 K for 1 h without sacrificing the magnetic properties, indicating a good balance between magnetic performance and mechanical property. The underlying mechanism is also discussed.

## 2. Materials and Methods

The ingots of the Fe_24.94_Co_24.94_Ni_24.94_Al_24.94_Si_0.24_ HEA were prepared using the arc-melting raw materials of Fe (purity 99.99%), Co (99.999%), Ni (99.995%), Al (99.99%) and Si (99.99%) by arc melting under an argon atmosphere. To guarantee compositional uniformity, each ingot was melted repeatedly at least four times. Then, the ingots were cast into bulk samples with dimensions of 100 (length) × 10 (width) × 2 (thickness) mm^3^ by water-cooled copper mold suction casting. Thermal analysis of the as-cast HEA is performed using differential scanning calorimetry (DSC, NETZSCH STA449F3, Bavaria, Germany) at a heating rate of 20 K/min. The samples were annealed at 573 K, 673 K and 873 K for 1 h in a vacuum furnace under a vacuum of 5 × 10^−3^ Pa. The magnetic properties at room temperature were determined by a vibrating sample magnetometer (VSM, Lake Shore 7407, Westerville, OH, USA) with the applied magnetic field of 0.5 T (about 398 kA/m). The mechanical properties were measured at room temperature by the nanoindentation (Hysitron TI980, Bruker Nano GmbH, Hamburg, Germany) under a load of 10 mN with a holding time of 5 s. The Berkovich diamond tip was used and ten points were measured on each sample. X-ray diffraction (XRD) patterns were collected using an Empyrean diffractometer (PANalytical, Almelo, The Netherlands) equipped with Cu Kα radiation (λ = 1.5406 Å). The Rietveld refinement method was employed to quantify the phase composition and analyze phase changes after the annealing treatment, using the JADE 6.0 software. The standard PDF cards referenced for each phase are as follows: BCC phase—ICDD PDF #37–0474 and B2 phase—ICDD PDF #44–1185. All PDF cards were retrieved from the International Centre for Diffraction Data (ICDD) PDF–4+ database, which ensures the accuracy and authority of the diffraction data used for refinement. The refinement parameters (R < 5%) indicated good agreement between the calculated and experimental XRD patterns, confirming the reliability of the phase quantification results. The scanning angle was from 20° to 100° and the scanning rate was 1° per minute. The microstructure was characterized by an SU8700 scanning electron microscope (SEM, JEOL Ltd., Tokyo, Japan) with an e–FlashFS backscatter diffraction detector (EBSD) (Bruker Nano GmbH, Hamburg, Germany). The EBSD samples were ground using 2000-grit SiC abrasive papers, followed by electrolytic polishing at 30 V for 50 s in a solution of 90% ethanol and 10% HClO_4_. The TEM observation was carried out by a transmission electron microscope equipped with an energy-dispersive (EDS) detector (TEM, JEOL Ltd., Tokyo, Japan). The samples for TEM were prepared by a focused ion beam (FIB, Helios G4 UX, Thermo Fisher Scientific, Waltham, MA, USA). Prior to FIB milling, a 100 nm-thick Pt protective layer was deposited on the surface of the samples to minimize Ga ion-induced damage during the milling process. The final electron-transparent region of the TEM lamellae was controlled to a thickness of 80–100 nm to ensure high-resolution imaging and accurate microstructural characterization.

## 3. Results

### 3.1. Magnetic and Mechanical Properties

**Figure 1:**
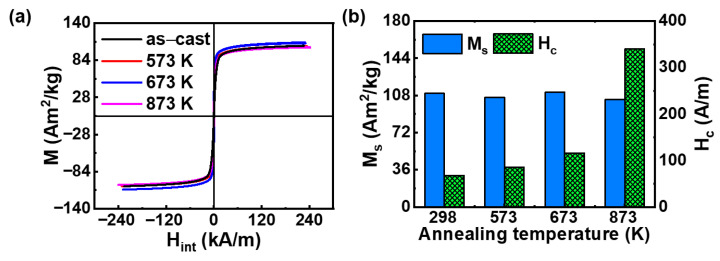
(**a**) The hysteresis loops and (**b**) the comparison of the M_s_ and the H_c_ in the as-cast and annealed Fe_24.94_Co_24.94_Ni_24.94_Al_24.94_Si_0.24_ HEAs.

Figure 1a shows the hysteresis loops of the as-cast and annealed Fe_24.94_Co_24.94_Ni_24.94_Al_24.94_Si_0.24_ HEA. The soft magnetic behavior is exhibited in all samples. The M_s_ and the H_c_ as a function of annealing temperature at room temperature are seen in Figure 1b. The M_s_ of the as-cast sample (298 K) is 110 Am^2^/kg, and it slightly changes after annealing at 573 K, 673 K and 873 K, see Table 1. In contrast, the H_c_ is increased after annealing and achieves the maximum (340 A/m) in the annealed HEA at 873 K. Notably, the annealed HEA at 573 K shows similar soft magnetic properties with the as-cast HEA, while obvious change is observed in the HEAs after annealing at 673 K and 873 K. Hence, we selected as-cast samples and HEAs annealed at 673 K and 873 K for further mechanical property characterization and microstructural analysis, aiming to elucidate the correlation between microstructure and properties.

**Figure 2:**
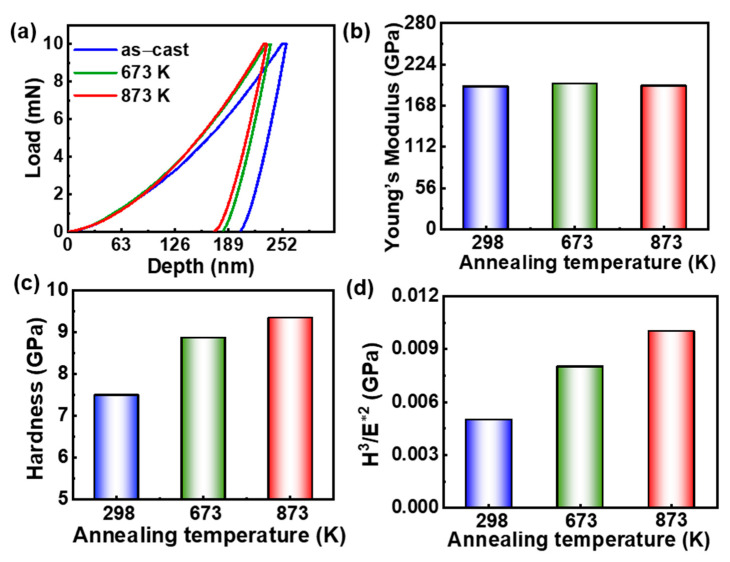
The nanoindentation result of the as-cast and annealed Fe_24.94_Co_24.94_Ni_24.94_Al_24.94_Si_0.24_ HEAs. (**a**) The load–depth (p–h) curves; (**b**) Young’s modulus; (**c**) hardness; and (**d**) the value of H^3^/E*^2^.

The nanoindentation load–depth (p–h) curves of the as-cast sample and annealed HEAs at 673 K and 873 K are shown in Figure 2a. The hardness and Young’s modulus can be obtained from Figure 2a and are listed in Table 1. The Young’s modulus exhibits a marginal change, from 194.0 ± 4.9 GPa of the as-cast HEA to 195.1 ± 5.8 GPa of the annealed HEA at 873 K, see Figure 2b. The hardness of the as-cast HEA is 7.49 ± 0.29 GPa, and it increased to 8.86 ± 0.43 GPa after annealing at 673 K, and 9.33 ± 0.47 GPa of the sample annealed at 873 K, indicating that the annealing treatment can improve the hardness of the Fe_24.94_Co_24.94_Ni_24.94_Al_24.94_Si_0.24_ HEA, see Figure 2c. The resistance to plastic deformation can be determined from the ratio of the hardness and effective Young’s modulus (H^3^/E*^2^) [33]. The E* is given by E* = E/(1 − ν), where E and ν are the Young’s modulus and Poisson ratio [34]. In comparison to the H^3^/E*^2^ of the as-cast sample (0.005), the annealed HEAs show higher H^3^/E*^2^, see Figure 2d. The maximum H^3^/E*^2^ (0.010) is achieved after annealing at 873 K, suggesting that the annealed HEA at 873 K exhibits the strongest resistance to plastic deformation.

### 3.2. Microstructure

**Figure 3:**
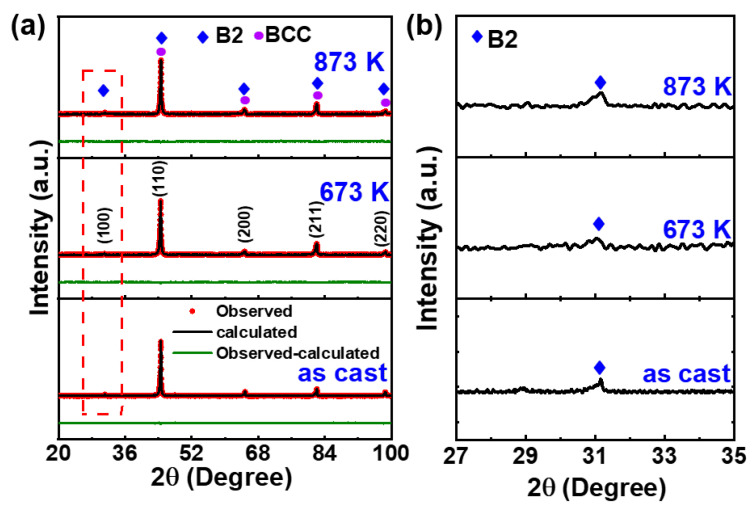
(**a**) The XRD patterns of the as-cast and annealed Fe_24.94_Co_24.94_Ni_24.94_Al_24.94_Si_0.24_ HEAs. The XRD data are analyzed by the Rietveld refinement. The experimental data are represented by red dots, and the calculated result is marked as a black solid line. The green solid line represents the difference between the experimental data and calculated data. (**b**) The enlarged view of a red dashed-line rectangle in (**a**).

XRD analysis of the patterns in Figure 3a reveals that all HEAs possess a dual-phase structure comprising BCC and B2 phases, indicating no alteration in phase constitution after the annealing process. Figure 3b is the enlarged view of the red dashed-line rectangle in Figure 3a, exhibiting a weak diffraction peak corresponding to the (100) peak of the B2 phase at 2θ = 31°. The lattice parameters of the BCC (a_BCC_) and B2 phases (a_B2_) are obtained by the Rietveld refinement of XRD data and are listed in Table 2. The a_BCC_ of the as-cast sample is 2.8726 ± 0.0005 Å, and it is slightly decreased to 2.8668 ± 0.0007 Å at 673 K and 2.8671 ± 0.0008 Å at 873 K. The a_B2_ marginally changes from 2.8739 ± 0.0004 Å of the as-cast HEA to 2.8734 ± 0.0006 Å of the annealed HEA at 673 K, and it is increased to 2.8811 ± 0.0009 Å after annealing at 873 K. At 673 K, the moderate atomic diffusion enables optimized atomic packing in the B2 phase, leading to a slight reduction in its lattice parameter. In contrast, annealing at 873 K enhances the segregation of Al and Ni atoms and induces a thermal expansion effect, both of which contribute to the increased a_B2._ This temperature-dependent variation in the B2 phase’s lattice parameter directly influences the lattice misfit between the B2 precipitates and the BCC matrix. The lattice misfit (ε) can be calculated by the formula ε = 2 × (a_B2_ − a_BCC_)/(a_B2_ + a_BCC_) [35,36]. The as-cast HEA shows small value of ε (0.023%); however, the annealed HEAs exhibit larger ε with 0.227% at 673 K and 0.244% at 873 K, which indicates that the annealing treatments lead to the increase in ε.

**Figure 4:**
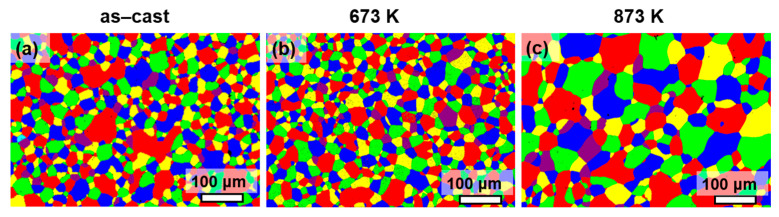
The grain distribution of the as-cast and annealed Fe_24.94_Co_24.94_Ni_24.94_Al_24.94_Si_0.24_ HEAs. (**a**) As-cast HEA; (**b**) the HEA annealed at 673 K; and (**c**) the HEA annealed at 873 K.

Figure 4 presents the EBSD images of the as-cast and annealed HEAs. The as-cast sample exhibits a relatively uniform grain distribution, with a grain size ranging from 4 μm to 75 μm and an average grain size of 30 μm. After annealing at 673 K, the HEA shows a more homogeneous grain distribution, corresponding to a grain size range of 5 μm to 70 μm and an average grain size of 34 μm. When the annealing temperature is elevated to 873 K, the sample displays a distinctly inhomogeneous grain distribution, characterized by the coexistence of coarse and fine grains; its grain size spans 10 μm to 120 μm, with an average grain size of 56 μm. At 873 K, the accelerated grain boundary migration leads to selective grain growth. Grains with favorable orientations grow rapidly, while fine grains are pinned by B2 nanoprecipitates, resulting in the coexistence of coarse and fine grains and thus a more inhomogeneous grain distribution compared with the as-cast and 673 K annealed samples.

**Figure 5:**
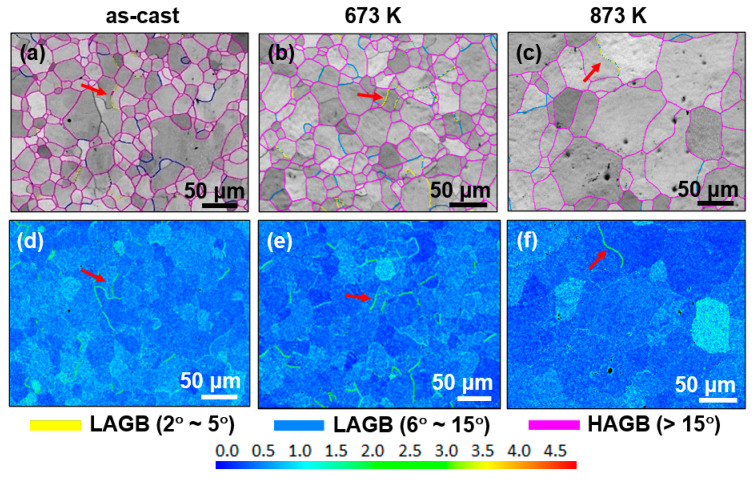
The EBSD images of the as-cast and annealed Fe_24.94_Co_24.94_Ni_24.94_Al_24.94_Si_0.24_ HEAs. (**a**–**c**) The grain-boundary character distribution. The HAGBs are marked in pink, LAGBs (2–5°) and LAGBs (6–15°) are identified in yellow and blue. (**d**–**f**) The kernel average misorientation (KAM) images. The color reference represents that the concentration of the strain is increased from blue to red.

The grain-boundary character distributions are shown in Figure 5a–c. Grain boundaries with a misorientation angle exceeding 15° are defined as high angle boundaries (HAGBs) and are marked in pink. The low angle boundaries (LAGBs) are those grain boundaries with misorientation angles ranging from 2 to 5° (identified in yellow) and 6–15° (blue color). The length fractions of LAGBs and HAGBs are determined from the Brandon criterion [37]. The fraction of LAGBs (2–5°) and LAGBs (6–15°) of the as-cast sample is 2.7% and 7.4%. It is changed to 2.6% and 9.6% of annealed HEA at 673 K and 1.0% and 6.4% of annealed HEA at 873 K. The proportion of HAGBs in the as-cast and annealed HEAs are 89.9%°, 87.8% and 92.5%, respectively, indicating that the HAGBs are dominant in all studied HEAs. The kernel average misorientation (KAM) can give an estimation of the strain distribution through measuring the local misorientation [38]. The KAM images are shown in Figure 5d–f, revealing that the strain concentration is low in all samples. The strain concentration is relatively higher at LAGBs (2–5°) than that in the grains, see the red arrows in Figure 5.

**Figure 6:**
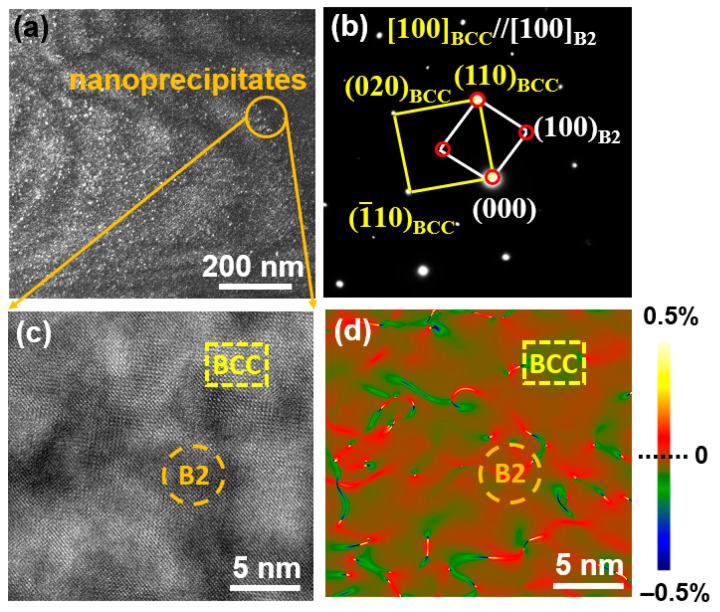
The TEM images of as-cast Fe_24.94_Co_24.94_Ni_24.94_Al_24.94_Si_0.24_ HEA. (**a**) The dark-field TEM image. The orange circle represents the region for HRTEM images. (**b**) The selected area electron diffraction (SAED) pattern. It confirms that the B2 nanoprecipitates are coherent with the BCC matrix. The yellow solid line indicates the SAED pattern of BCC matrix, and the white solid line and red circles represent the SAED patterns of B2 phase. (**c**) The HRTEM image from the orange circle in (**a**). The yellow dashed-line rectangle and orange dashed-line circle represent the BCC phase and B2 phase respectively. (**d**) The geometric phase analysis (GPA) image of strain distribution. The positive and negative values suggest tensile strain (from red to yellow) and compressive strain (from green to blue), respectively.

Figure 6a shows the dark-field (DF) image of the as-cast Fe_24.94_Co_24.94_Ni_24.94_Al_24.94_Si_0.24_ HEA, and spherical nanoprecipitates can be observed. The selected area electron diffraction (SAED) pattern is indexed as BCC and B2 structures along the [100] zone axis, see Figure 6b. Moreover, a coherent relationship is exhibited between the B2 precipitates and the BCC matrix. According to the high-resolution TEM (HRTEM) image in Figure 6c, the average size of the spherical nanoprecipitate is approximately 5 nm. Figure 6d shows the geometric phase analysis (GPA), which reveals a visualization of in-plane strain distribution [39]. The positive value represents the tensile strain while the negative value refers to compressive strain. The tensile strain is distributed in B2 precipitates while the compressive strain is observed in the BCC matrix, see Figure 6d.

**Figure 7:**
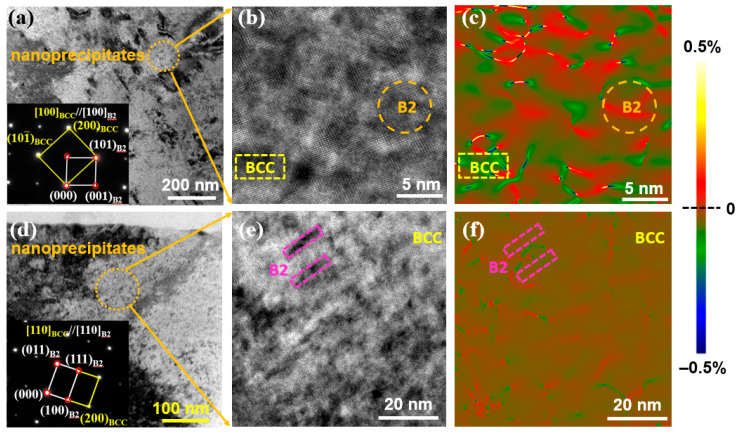
(**a**–**c**) Bright-field TEM, HRTEM and GPA images of Fe_24.94_Co_24.94_Ni_24.94_Al_24.94_Si_0.24_ HEA annealed at 873 K along the [100] zone axis. The inset of (**a**) is the SAED patterns. It confirms that the B2 precipitates are coherent with the BCC matrix. The HRTEM image from the orange dotted-line circle in (**a**) is enlarged as the HRTEM image in (**b**). The orange dashed-line circle and yellow dashed-line rectangle in (**b**,**c**) represent the spherical nanoprecipitate and the BCC matrix, respectively. (**d**–**f**) Bright-field TEM, HRTEM and GPA images of Fe_24.94_Co_24.94_Ni_24.94_Al_24.94_Si_0.24_ HEA annealed at 873 K along the [110] zone axis. The HRTEM image from the orange dotted-line circle in (**d**) is enlarged as the HRTEM image in (**e**). The pink dashed-line rectangle in (**e**,**f**) represent the rod-shaped nanoprecipitates.

The TEM results of the Fe_24.94_Co_24.94_Ni_24.94_Al_24.94_Si_0.24_ HEA annealed at 873 K are shown in Figure 7a–c. The spherical B2 nanoprecipitates are coherent with the BCC matrix by indexing the SAED pattern along the [100] zone axis (see the inset of Figure 7a). The orange dotted-line circle is chosen in Figure 7a and enlarged as HRTEM images in Figure 7b. The average size of spherical nanoprecipitates is approximately 6 nm. The B2 nanoprecipitates exhibit the distribution of tensile strain while the BCC matrix shows compressive strain, see GPA image in Figure 7c. It is worth noting that the rod-shaped B2 nanoprecipitates, uniformly distributed within the BCC matrix, are observed along the [110] zone axis (Figure 7d). These B2 nanoprecipitates are also coherent with the BCC matrix, see the inset of Figure 7d. The HRTEM image in Figure 7e is the enlarged view of the dotted-line circle in Figure 7d. The rod-shaped B2 nanoprecipitate has a size of 15 nm in length and 4 nm in width (marked in pink dashed-line rectangle). Compared with the strain distribution in the spherical B2 nanoprecipitates and BCC matrix in Figure 7c, the level of strain distribution is reduced in the rod-shaped B2 nanoprecipitates and BCC matrix (Figure 7f) due to the released stress during the annealing treatment.

It is worth noting that the increased ε after annealing does not contradict the reduced strain concentration observed in the KAM (Figure 5) and GPA (Figure 7) results. ε is an intrinsic parameter reflecting the geometric mismatch between the BCC matrix and B2 precipitates, which increases due to annealing-induced changes in the phase lattice parameters (Table 2). In contrast, KAM quantifies the local plastic strain from residual dislocations and grain boundary misorientations, which is relieved by annealing (e.g., dislocation annihilation and grain boundary stabilization). GPA results further confirm that the elastic strain around precipitates is reduced by the formation of rod-shaped B2 precipitates at 873 K—these precipitates align along the low-strain [110] direction of the BCC matrix, releasing the elastic stress accumulated around spherical precipitates in the as-cast state. Thus, the increased lattice misfit and reduced strain concentration are independent consequences of annealing: the former reflects the phase lattice parameter evolution, while the latter reflects strain relaxation through microstructural optimization.

**Figure 8:**
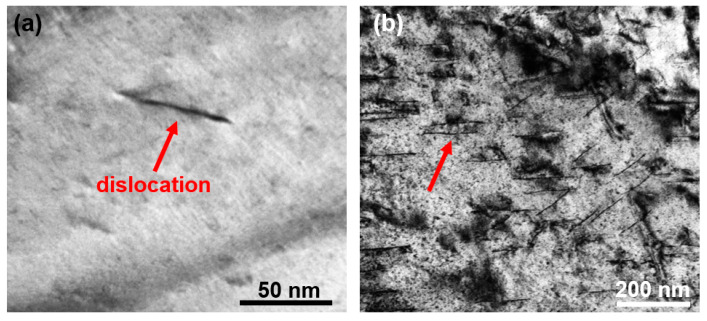
The dislocations (marked as red arrows) are observed in TEM images. (**a**) As-cast Fe_24.94_Co_24.94_Ni_24.94_Al_24.94_Si_0.24_ HEA; and (**b**) the annealed HEA at 873 K.

**Figure 9:**
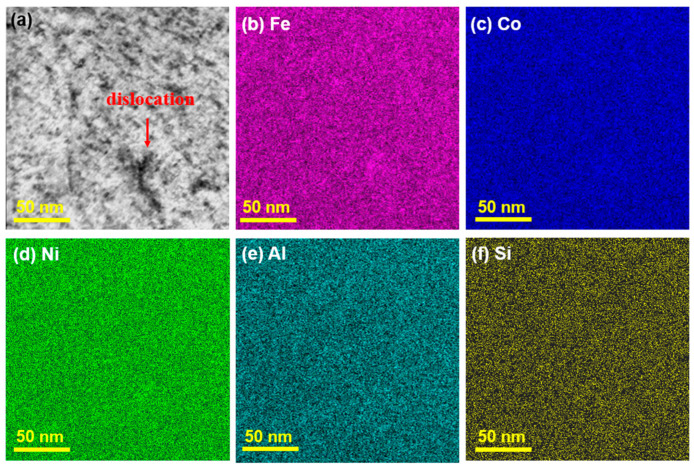
STEM images of Fe_24.94_Co_24.94_Ni_24.94_Al_24.94_Si_0.24_ HEA annealed at 873 K: (**a**) HADDF image and (**b**–**f**) elemental mapping images.

As indicated by the red arrows, dislocations are detected in two types of samples: the as-cast specimen (Figure 8a) and the HEA subjected to annealing at 873 K (Figure 8b). Compared with the as-cast HEA, the density of dislocations in the annealed HEA dramatically increases. The increased dislocation density is attributed to the stress induced by the precipitation of rod-shaped B2 nanoprecipitates. Specifically, the increased lattice misfit between the B2 precipitates and the BCC matrix gives rise to substantial elastic stress. This stress further promotes the formation of dislocations as a stress-relief mechanism, which offsets the dislocation annihilation effect that typically occurs during annealing. Consequently, the annealed sample exhibits a higher dislocation density than the as-cast sample. Figure 9 shows the STEM images of the annealed HEA at 873 K. All elemental distribution in Figure 9b–f is uniform, including the dislocation (red arrow in Figure 8a), indicating that the presence of nanoprecipitates and dislocations does not cause obvious elemental segregation.

## 4. Discussion

### 4.1. The Magnetic Mechanism

From the above results, it can be observed that the annealing treatment induces a slight variation in the M_s_ of the Fe_24.94_Co_24.94_Ni_24.94_Al_24.94_Si_0.24_ HEA, whereas the H_c_ increases remarkably. This phenomenon is evidently closely associated with the microstructural evolution of the alloy.

The saturation magnetization, M_s_, is an intrinsic magnetic parameter. That is, it is sensitive to the alloy’s composition and phase constitution. The TEM results in Figure 6 and Figure 7 show that the average size of spherical B2 nanoprecipitates is 5 nm in the as-cast Fe_24.94_Co_24.94_Ni_24.94_Al_24.94_Si_0.24_ HEA, and it keeps a similar value (6 nm) in the HEA annealed at 873 K. It indicates that the spherical B2 nanoprecipitate is stable and the coarsening is hindered. Furthermore, rod-shaped B2 nanoprecipitates (15 nm in length, 4 nm in width) are observed in the alloy after annealing at 873 K. The slight variation in the saturation of M_s_, induced by the annealing treatment, can be attributed to the precipitation of rod-shaped nanoprecipitates within the alloy.

The coercivity, H_c_, is an extrinsic magnetic parameter that is sensitive to the microstructure [40]. The grain size (D) has a significant effect on the soft magnetic property. The relationship of H_c_ ∝ 1/D is followed when the grain size is micrometer scale [41]. In this study, the average grain size is 30 μm for as-cast HEA, 34 μm and 56 μm for the annealed HEA annealed at 673 K and 873 K. However, the respective H_c_ of the three samples is 68 A/m, 116 A/m and 340 A/m, which fails to obey the 1/D law. It indicates that other influencing factors on the H_c_ should be considered_._ The change in the H_c_ can be understood from three aspects. (1) The uniformity of grain distribution: After the alloy is annealed at 873 K, it exhibits the characteristic of coexisting coarse grains and fine grains (see Figure 4), that is, a significantly inhomogeneous grain distribution. This is unfavorable for the rotation of magnetic moments during the demagnetization process, resulting in an increase in the H_c_ of the alloy. (2) The increase in the lattice misfit: When the misfit between the nanoprecipitate and the matrix is small, the lattice near the phase interface is only slightly distorted, resulting in a weak internal stress field. This weak internal stress field fails to hinder the movement of the magnetic domain walls during magnetization [40]. When the misfit between the nanoprecipitate and the matrix is large, the high stress field generated near the interface will significantly hinder the movement of the domain walls, thereby increasing H_c_ [42]. In the current study, the lattice misfit increases to 0.227% of the annealed HEA at 673 K and to 0.244% at 873K compared with the lattice misfit of the as-cast HEA (0.023%), leading to the increase in the H_c_ of annealed HEAs. (3) The presence of high-density dislocations: The dislocations can act as pinning centers for magnetic domain wall motion, which results in the increase in H_c_ [43,44]. In this work, a higher density of dislocations is observed in the HEA annealed at 873 K (Figure 8).

### 4.2. Strengthening Mechanism

The dramatical increase in hardness (9.33 ± 0.47 GPa) is shown in the annealed HEA at 873 K, which is 25% higher than that of the as-cast HEA. Hardness is closely correlated with yield strength (σ_0.2_), which can be calculated using the following formula [45].

*H* = *k σ*_0.2_ (1)

where k is the empirical coefficient and is given as 3 [46].

It is attributed to the combined effect of multiple strengthening mechanisms: mainly a combination of dislocation strengthening (σ_d_) and precipitation strengthening (σ_p_). Therefore, the increment of the yield strength of the HEA can be calculated according to the following formula [47,48,49]:

*Δσ*_0.2_ = *Δσ_d_* + *Δσ_p_* (2)

The contribution of dislocation strengthening is given by the Bailer–Hirsch formula [50]:

(3) $$\sigma_{d}\;=\;M\alpha Gb\rho^{1/2}$$

where M is the Taylor factor and G is the shear modulus. The α is a constant (0.4) for BCC crystals [51], and the b is the Burgers vector. Here b = 0.2483 nm for BCC matrix that is calculated by b = a/2 <1 1 1>. The ρ is the dislocation density that is given as 8.73 × 10^13^ m^−2^ from the TEM image. Hence, the calculated σ_d_ is 188 MPa in the annealed HEA at 873 K. Because the dislocation density of as-cast can be ignored (see Figure 8a), compared with the as-cast HEA, the increment of σ_d_ is 188 MPa in the annealed HEA at 873 K.

Although the shape and size of the B2 nanoprecipitates in the annealed HEA at 873 K are changed, the B2 nanoprecipitates are still coherent with the BCC matrix, indicating that the dislocations can be impeded by coherent precipitates by a shearing mechanism [52]. Hence, the σ_p_ is mainly from coherency strengthening (σ_coherency_), modulus strengthening (σ_modulus_) and order strengthening (σ_order_) [53]. The calculation formulas are shown as follows [54]:

(4) $$\sigma_{order}\;=\;0.81M(\frac{\gamma_{APB}}{2b}){\left(\frac{3\pi f}{8}\right)}^{1/2}$$

(5) $$\sigma_{modulus}=0.0055M{\left(∆G\right)}^{\frac{3}{2}}{\left(\frac{2f}{G}\right)}^{1/2}{\left(\frac{r}{b}\right)}^{3m/2-1}$$

(6) $$\sigma_{coherency}=\;M\alpha {\left(G\varepsilon \right)}^{3/2}{\left(\frac{rf}{0.5Gb}\right)}^{1/2}$$

where M is the Taylor factor, b is the Burgers vector of the matrix, γ_APB_ is the antiphase boundary energy, f is the volume fraction of precipitates, G is the modulus of the matrix, ΔG is the difference in the shear modulus between the matrix and precipitates and r is the average radius of the sphere precipitates. The respective m and α are a constant, being 0.85 and 2 [32]. γ_APB_ is estimated as 200 mJ/m^2^ for the precipitates in the BCC phase [55]. ΔG is 3 GPa for the BCC matrix [56]. The calculated σ_order_, σ_modulus_ and σ_coherency_ are 1014 MPa, 26 MPa and 242 MPa. The value of σ_order_ is greater than (σ_coherency_ + σ_modulus_), indicating that the main contribution of precipitation strengthening comes from order strengthening. Our previous work reported that σ_order_ is 545 MPa in the as-cast HEA [32]. Hence, compared with the as-cast HEA, the increment of σ_order_ is 469 MPa in the annealed HEA at 873 K.

Therefore, the yield strength increment of the HEA annealed at 873 K is the sum of the increment σ_d_ (188 MPa) and increment σ_order_ (469 MPa), reaching a value of 657 MPa. In contrast, the yield strength increment estimated from the alloy’s hardness difference (see Table 1) using Formula (1) is 613 MPa. These two values are in good agreement.

**Figure 10:**
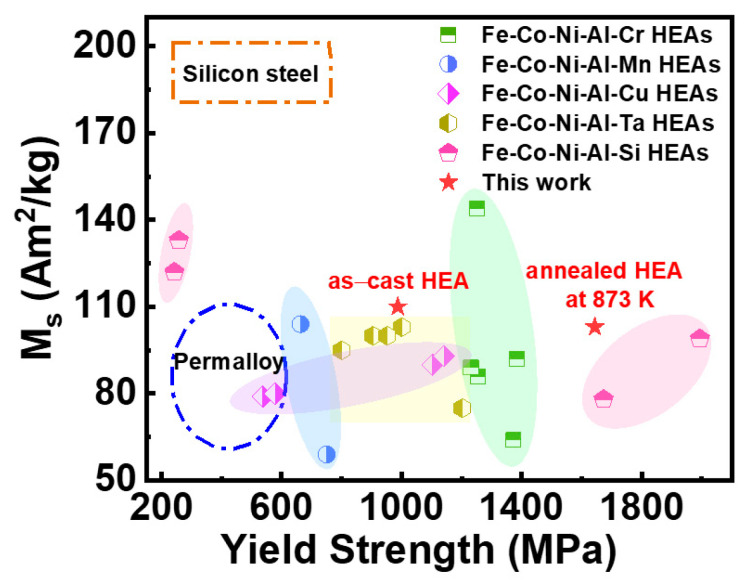
The comparison of M_s_ and yield strength among our work, some reported ferromagnetic HEAs, and conventional soft magnetic materials (e.g., Permalloy and Silicon steel). The studied HEAs in this work are marked as red pentagrams.

Figure 10 compares the M_s_ and yield strength of the present work (marked by red pentagrams) with those of several reported typical SMMs and ferromagnetic HEAs [10,16,32,57,58]. Zhang et al. found that the high M_s_ (122–133 Am^2^/kg) and the low yield strength (242–257 MPa) can be obtained in the FeCoNi(AlSi)_x_ (x = 0.1, 0.2) HEAs. In contrast, the FeCoNi(AlSi)_x_ (x = 0.4, 0.5) HEA exhibits a significantly high yield strength (1673–1994 MPa) and a relatively low M_s_ (78–99 Am^2^/kg) [10]. Compared with these studies, an excellent yield strength (987–1643 MPa) and M_s_ (103–113 Am^2^/kg) can be observed in this work simultaneously. It suggests that the studied Fe_24.94_Co_24.94_Ni_24.94_Al_24.94_Si_0.24_ HEA in our work has a combination of good magnetic and mechanical properties.

## 5. Conclusions

In this study, the microstructure and soft magnetic and mechanical properties of the Fe_24.94_Co_24.94_Ni_24.94_Al_24.94_Si_0.24_ HEA after annealing at different temperatures were systematically investigated. The main conclusions are summarized as follows.

The as-cast HEA is composed of a BCC matrix phase and spherical B2 nanoprecipitates with a diameter of approximately 5 nm, and a coherent relationship exists between the B2 phase and the BCC matrix. After the annealing treatment at 873 K, the alloy still consists of both BCC and B2 phases, with their coherent relationship maintained; in addition to the spherical B2 nanoprecipitates, rod-shaped B2 nanoprecipitates can also be observed, which also exhibit a coherent relationship with the matrix. The annealing treatment increases the lattice misfit of the alloy, which increases from 0.023% in the as-cast sample to 0.244% in the HEA annealed. After annealing at 873 K, the H_c_ increases and reaches a maximum value of 340 A/m, which is attributed to the inhomogeneous grain distribution, increased lattice misfit and high dislocation density. The hardness is dramatically increased after annealing at 873 K; it is 25% higher than that of as-cast HEA. It comes from multiple strengthening mechanisms, and the order strengthening is the main contribution.

## Figures and Tables

**Table 1:** The magnetic and mechanical properties of the as-cast and annealed Fe_24.94_Co_24.94_Ni_24.94_Al_24.94_Si_0.24_ HEAs.

HEA	M_s_ (Am^2^/kg)	H_c_ (A/m)	Hardness (GPa)	Young’s Modulus (GPa)	H^3^/E*^2^ (GPa)
as-cast	110	68	7.49 ± 0.29	194.0 ± 4.9	0.005
573 K	105	86	–	–	–
673 K	113	116	8.86 ± 0.43	197.8 ± 5.2	0.008
873 K	103	340	9.33 ± 0.47	195.1 ± 5.8	0.010

**Table 2:** The phase constitutions and lattice parameters of the as-cast and annealed Fe_24.94_Co_24.94_Ni_24.94_Al_24.94_Si_0.24_ HEAs.

Sample	Phase	a_BCC_ (Å)	a_B2_ (Å)	ε (%)
as-cast	BCC + B2	2.8726 ± 0.0005	2.8739 ± 0.0004	0.023
673 K	BCC + B2	2.8668 ± 0.0007	2.8734 ± 0.0006	0.227
873 K	BCC + B2	2.8671 ± 0.0008	2.8811 ± 0.0009	0.244

## Data Availability

The data presented in this study are available upon request from the corresponding authors.
